# Integration of Radiomic and Multi-omic Analyses Predicts Survival of Newly Diagnosed IDH1 Wild-Type Glioblastoma

**DOI:** 10.3390/cancers11081148

**Published:** 2019-08-10

**Authors:** Ahmad Chaddad, Paul Daniel, Siham Sabri, Christian Desrosiers, Bassam Abdulkarim

**Affiliations:** 1 Division of Radiation Oncology, Department of Oncology, McGill University, Montreal, QC H4A 3J1, Canada; 2 The Laboratory for Imagery, Vision and Artificial Intelligence, École de Technologie Supérieure (ETS), Montréal, QC H3C 1K3, Canada; 3 Department of Pathology, McGill University, Montreal, QC H4A 3J1, Canada; 4 Research Institute of the McGill University Health Centre, Glen Site, Montreal, QC H4A 3J1, Canada

**Keywords:** IDH1, radiomics, glioblastoma, survival time

## Abstract

Predictors of patient outcome derived from gene methylation, mutation, or expression are severely limited in IDH1 wild-type glioblastoma (GBM). Radiomics offers an alternative insight into tumor characteristics which can provide complementary information for predictive models. The study aimed to evaluate whether predictive models which integrate radiomic, gene, and clinical (multi-omic) features together offer an increased capacity to predict patient outcome. A dataset comprising 200 IDH1 wild-type GBM patients, derived from The Cancer Imaging Archive (TCIA) (n = 71) and the McGill University Health Centre (n = 129), was used in this study. Radiomic features (n = 45) were extracted from tumor volumes then correlated to biological variables and clinical outcomes. By performing 10-fold cross-validation (n = 200) and utilizing independent training/testing datasets (n = 100/100), an integrative model was derived from multi-omic features and evaluated for predictive strength. Integrative models using a limited panel of radiomic (sum of squares variance, large zone/low gray emphasis, autocorrelation), clinical (therapy type, age), genetic (CIC, PIK3R1, FUBP1) and protein expression (p53, vimentin) yielded a maximal AUC of 78.24% (*p* = 2.9 × 10^−5^). We posit that multi-omic models using the limited set of ‘omic’ features outlined above can improve capacity to predict the outcome for IDH1 wild-type GBM patients.

## 1. Introduction

Glioblastoma (GBM, grade IV astrocytoma) is the most common and deadly brain tumor [1,2]. Median survival of 15 months has remained essentially unchanged since the introduction of trimodal therapy [3], which combines maximum safe resection, radiation therapy (RT), and systemic temozolomide (TMZ). GBM can be divided into two types: Primary GBM, which arises de novo, and secondary GBM, which is an evolutionary progression from low-grade glioma (LGG) [4]. Approximately 70% to 80% of secondary GBMs have mutations in the isocitrate dehydrogenase 1 (IDH1) gene that are absent in primary GBM [5,6].

The emergence of next-generation sequencing (NGS) technologies has allowed unprecedented characterization of the molecular landscape of glioma and stimulated the search for means of disease stratification and prediction of patient survival. The use of ‘omic’ analysis for identification of survival features has had the most success in grade II/III glioma where distinct subtypes of gliomas can be defined by combinations of mutations in isocitrate dehydrogenase (IDH1-R132H), telomerase (TERT) and 1p/19q chromosomal co-deletion [7]. Similar efforts to characterize GBM have revealed that, whilst several molecular subtypes exist [4,8], only the glioma-CpG island methylated phenotype (G-CIMP), which harbor the IDH1-R132H mutation, is predictive of longer patient survival [9]. Additional predictors of survival for patients with wild-type IDH1 are lacking and largely limited to MGMT promoter methylation, highlighting the need for alternate means of patient stratification. 

Routine magnetic resonance (MR) imaging captures the physical properties of the entire tumor volume in considerable detail and is routinely used in the clinic to define disease progress, invasion, and multifocality. These characteristics guide treatment decisions including surgical resection, therapies and subsequent follow-up. Arguably, the true potential of MR imaging is underutilized in the clinic given the recognition that volumetric and quantitative (‘radiomic’) features extracted from MR scans reflect various biological aspects of the disease, that have potential diagnostic and prognostic relevance [10,11]. For example, textural features extracted from contrast enhancement, edema, and regions of necrosis have been related to survival in GBM [12,13,14,15]. Similarly, radiomics has been shown to be able to identify other diagnostically informative features such as O-6-methylguanine-DNA methyltransferase (MGMT) promoter methylation, various somatic mutations or activation of specific molecular pathways [16,17,18]. Additionally, MRI offers unprecedented insight into aspects of intra-tumor heterogeneity [19,20,21] and enables to define the spatial distribution of biological properties within a tumor (e.g., areas of high and low proliferation, invasion, etc). 

To date, there has been no comprehensive study highlighting the association between radiomics and biological or clinical features for IDH1 wild-type GBM. As such, we sought to evaluate the capacity of radiomics to discern underlying molecular features relevant to diagnosis and treatment. Furthermore, due to the capacity for various interrogative methods (i.e., radiomics, genomics, transcriptomics, proteomics) to identify diverse and often distinct aspects of the disease state, we hypothesized that an integrative model utilizing data from each ‘omic’ analysis would better predict patient survival than models using individual data sources. Notably, we focused upon defining a predictive signature for newly diagnosed IDH1 wild-type GBM to address the absence of prognostic biomarkers for these patients. 

## 2. Results

### 2.1. Patients Characteristics

In this study, we used a dataset of 200 patients with newly diagnosed GBM from the NCI’s Cancer Imaging Archive (TCIA, n = 71), and an internal cohort treated at McGill University Health Centre (MUHC, n = 129) between 2005 and 2012 and previously reported by our group [22] (Table 1). The median age of patients was 61 and 62 in TCGA and MUHC cohorts, respectively. In TCGA cohort, 71 patients underwent surgery, and 66 received adjuvant RT, with or without concomitant TMZ. From the MUHC cohort, all 129 patients with histologically-proven GBM received maximum safe resection. Fifteen patients did not receive RT, whilst 112 patients received adjuvant RT with or without concomitant TMZ. Seven patients (5 TCGA patients and 2 MUHC patients) had no record of treatment. All patients had accessible MRI data taken from the time of the first diagnosis. Median survival of the TCGA and MUHC cohort was 12.06 and 13.73 months, respectively (Table 1).

### 2.2. Assessment of Radiomic Features to Identify Tumor Characteristics

We used the Wilcoxon rank-sum test to compare the distribution of radiomic feature values across gene mutation or methylation status (e.g., EGFR, MGMT, etc.) and Kruskal–Wallis test to compare between radiomic features values across molecular subtypes (i.e., classical, mesenchymal, proneural, and neural) of IDH1 wild-type GBM patients (Figure S1). Considering TCGA patients (n = 63 for molecular subtypes, n = 37 for gene status), we found that several genes (i.e., EGFR, FGD5, PIK3C2G, PCLO) were significantly correlated to radiomic features prior to multiple-hypothesis correction. Following Holm–Bonferroni correction, radiomic features were not able to discriminate between molecular subtypes or MGMT methylation status. Increasing the availability of patients with genetic characterization (i.e., from 73 TCGA patients, only 37 had mutation data available) may allow greater resolution of the ability for radiomics to identify relevant genetic features in GBM.

### 2.3. Radiomic Signature to Predict Survival of IDH1 Wild-Type GBM Patients 

**Figure 1:**
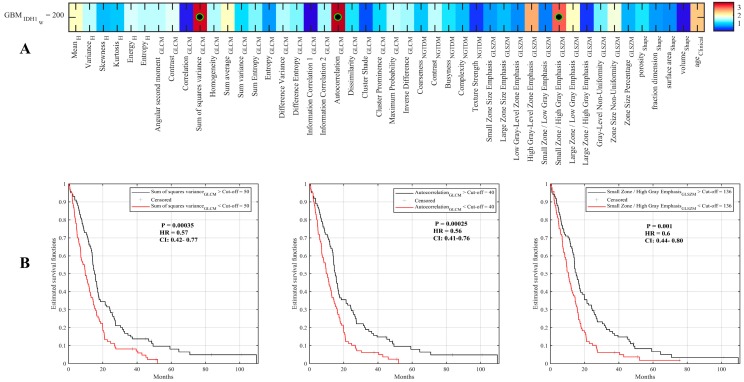
Univariate prediction of survival using radiomic features (**A**) Log-rank significance of survival difference between separated by individual radiomic features. (**B**) Kaplan–Meier survival curves for “autocorrelation”, “sum square variance” and “small zone/high gray emphasis”.

We sought to investigate whether individual radiomic features could predict IDH1 wild-type GBM patient survival. For this, we utilized the 45 radiomic features for 200 IDH1 wild-type GBM patients (71 from TCGA and 129 from MUHC, Figure 1A) and considered the median value for each feature to separate patients into groups (i.e., greater than median vs. less than median). The ability of each radiomic feature to predict survival was then evaluated using log-rank significance testing (Figure 1A). We found that, after Holm–Bonferroni correction [23], three radiomic features (sum square variance, autocorrelation and small zone/high gray emphasis) were able to predict the outcome of IDH1 wild-type GBM patients. Specifically, longer survival was associated with a higher feature value for sum square variance (HR = 0.57, CI: 0.42–0.77, 15.1 vs. 9.7 months), autocorrelation (HR = 0.56, CI: 0.41–0.76, 14.88 vs. 9.75 months) and small zone/high gray emphasis (HR = 0.5, CI: 0.44–0.8, 14.75 vs. 10.15 months) (Figure 1B). The three radiomic features represent the characteristics of images. For example, the Sum-square variance measures the local texture variation, autocorrelation describes the fineness and coarseness of the texture, and small zone/high gray emphasis describe the small zone of high grey-level (Table S1). Based on the median value of these texture features, it is able to distinguish between two survival groups, as shown in Figure 1B.

These three significant features describe the heterogeneity of contrast enhancement of GBM tumors (Table S2). Using FLAIR scans of the 71 patients from TCIA, we found no significant features to predict the outcome of IDH1 wild-type GBM (Figure S2A). 

### 2.4. Improved Prognostic Capacity When Integrating Radiomics with Genomic Features

**Figure 2:**
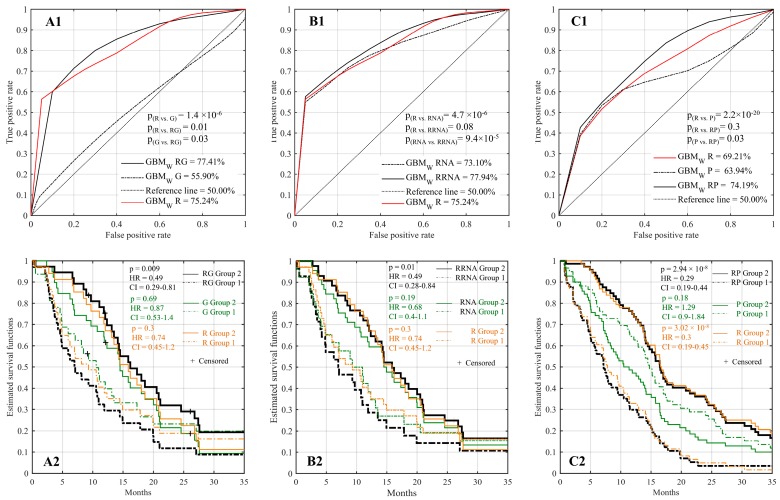
Multivariate prediction models integrating radiomics with genomic, transcriptomic or protein expression-immunohistochemistry (IHC) analysis. Using The Cancer Imaging Archive (TCIA)/The Cancer Genome Atlas (TCGA) datasets (n = 71), (**A1**) Area under the Receiver Operating Characteristic(ROC) curve of genomics alone (GBMw G), radiomics alone (GBMw R) or when using a combination of genetic and radiomic features (GBMw RG). (**B1**) Area under the ROC of the transcriptome alone (GBMw RNA), radiomics alone (GBMw R) or when using a combination of transcriptomic and radiomic features (GBMw RRNA). Kaplan–Meier estimate for the TCGA patient cohort when analyzed using the radiomics/genomics (**A2**) or radiomics/transcriptomic (**B2**) models. Using our internal McGill University Health Centre (MUHC) cohort (n = 129), (**C1**) Area under the ROC of either proteins alone (GBMw P), radiomics alone (GBMw R) or when combining radiomics with IHC features (GBMw RP). (**C2**) Kaplan–Meier survival of patients analyzed with the combined radiomics/IHC prediction model. Significance p-value derived from chi-square test (**A1, B1,** and **C1**) for comparing the predicted survival groups corresponding to AUCs.

Genomic analysis is now part of routine clinical practice following initial surgery. As such, we sought to look at whether integration of mutational data alongside radiomics offers a greater capacity to predict IDH1 wild-type GBM patient survival. Given the limited capacity for mutations to define patient cohorts with different survival times (Figure S3), we utilized the 100 most commonly mutated genes in GBM as described by TCGA (Table S4) to integrate into our predictive model [24]. Integration of genomic mutations with T1 derived radiomic features (GBM_w_ RG) improved prediction, achieving an AUC of 77.41% compared to 75.24% and 55.90% with radiomics (GBM_w_ R) or genomics (GBM_w_ G) alone, respectively (Figure 2A1). Notably, comparison of AUC obtained from combining genomics and radiomics to that of radiomics alone reached significance in a chi-square test (*p* = 0.01, Table S5), indicating additive value when integrating genomics into predictive models. Additionally, predictive model using the combined features was found to be significantly better than with genomics in predicting the survival group.

Similar results were found when using FLAIR in multivariate models (AUC = 72.01%, Figure S2B). Using the multivariate RF classifier model to partition patients to short-term and long-term survival groups, we applied the log-rank significance test to assess the feasibility of our integrated predictive model. We found that differential survival could be predicted when integrating T1 radiomics with genomic (*p* = 0.009, HR = 0.49, CI: 0.29–0.81, Figure 2A2) as well as FLAIR and genomic features (*p* = 0.008, HR = 0.45, CI: 0.25–0.79l, Figure S2C). Together, these demonstrate the utility of integrating limited genomic analysis to improve predictive models.

### 2.5. Improved Prognostic Capacity When Integrating Radiomics with Transcriptomic Features

In addition to genomic features, transcriptomic analysis is becoming more available for in-depth characterization of underlying tumor features, especially with the limited pricing for panels covering a limited gene set. Accordingly, we investigated whether expression data also provides benefit to predictive models when integrated alongside radiomics. To define a limited subset of genes from the whole genome to test in our predictive model, we first identified genes which are differentially expressed between short- and long-term survivors. Using TCGA patients with available transcriptomic analysis (n = 346), when separating patients based upon median expression of each gene then performing log-rank analysis testing, we found 498 genes which were differentially expressed between patients with short or long survival in univariate analysis (Table S3). Combining transcriptomic data with radiomics (GBM_w_ RRNA) improved AUC to 77.94% compared to AUC of 73.10% when using transcriptomic data (GBM_w_ RNA) alone (Figure 2B1), however this increase in AUC did not reach significance (*p* = 0.08, Table S5). Log-rank significance test showed that transcriptomic (p = 0.01, HR = 0.49, CI: 0.28–0.84, Figure 2B2) features integrated alongside radiomics could predict survival, again demonstrating the utility for limited expression analysis in this context.

### 2.6. Improved Prognostic Capacity When Integrating Radiomics with Protein Expression

Finally, we also sought to determine whether immunohistochemical (IHC) analysis of protein expression could improve prediction. IHC is a standard technique utilized in all clinics for routine analysis of tumor specimens. To evaluate predictive models integrating IHC analysis, we used IHC analysis of six proteins (i.e., Ki67, EGFR, PTEN, CD44, p53, vimentin) which include proteins routinely analyzed in current clinical practice, using a tissue microarray constructed for our internal MUHC cohort of GBM IDH1 wild-type patients (n = 129). We found that the AUC increased to 74.19% when integrating protein expression with radiomic features (GBM_w_ RP) compared to AUC of 63.94% and 69.21% using only protein expression or radiomic features, respectively (Figure 2C1), however this did not reach significance (*p* = 0.3, Table S5). The impact of integrating IHC analysis with radiomic features (i.e., combined features) on survival is outlined in Figure 2C2. These results show that differential survival can be predicted when combined radiomic with IHC features (*p* = 2.94 × 10^−8^, HR = 0.29, CI: 0.19–0.44). Using our internal dataset, we found that radiomics alone was able to predict survival (*p* = 3.02 × 10^−8^, HR = 0.3, CI: 0.19–0.45), in contrast to the TCGA dataset. However, using IHC analysis of these proteins was not predictive of IDH1 wild-type GBM patient survival. 

### 2.7. Multi-Omic Integrative Model and Identification of Predictive Features

**Figure 3:**
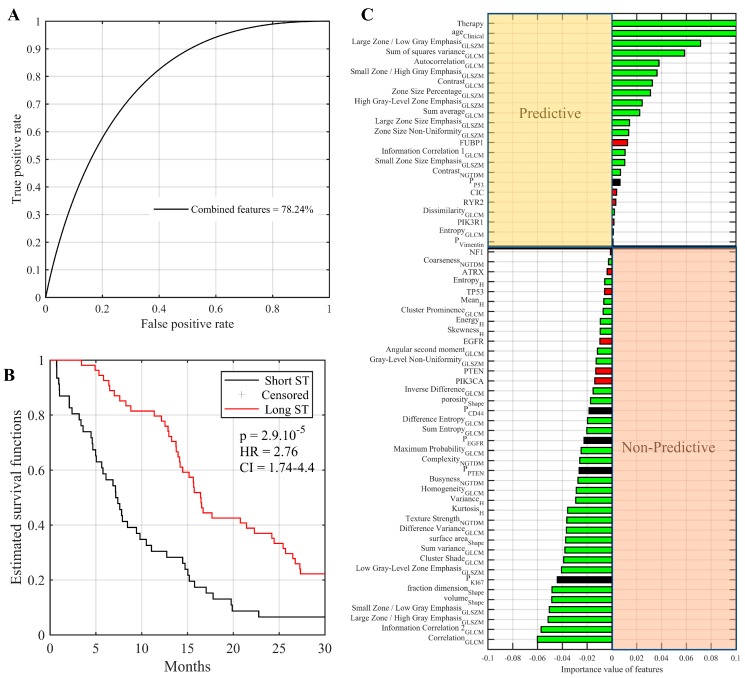
Validation of omics model for predicting the OS groups (i.e., short and long survival outcome) with IDH1 wild type GBM patients using 100 patient datasets for training and 100 for test (**A**) Area under the ROC of combined features derived from radiomics, genomics, IHC, and transcriptomics for predicting OS groups. (**B**) Kaplan–Meier curve of the patients’ groups that predicted their OS by omics model. (**C**) Importance of individual features for predicting OS groups (i.e., short and long survival outcome) using the RF classifier. Reported values correspond to the average increase in prediction error obtained by permuting the values of individual features across out-of-bag observations [25]. Positive and negative values correspond respectively to predictive and non-predictive features.

Given the change in AUC obtained from combining radiomics with different ‘omic’ datasets, we then assessed if integrating all radiomics, genomics, transcriptomics and IHC features in a single model would lead to further improvements. For this analysis, we randomly assigned the 200 GBM patients with IDH1 wild type into training (n = 100) or testing (n = 100) datasets. Using the test dataset (n = 100), we found that combining radiomics, genomic, IHC and transcriptomics along with clinical variables (with age and therapy type) increased AUC-ROC value to 78.24%, corresponding to a moderate improvement over models built from radiomics and single ‘omic’ analysis (Figure 3A). Kaplan–Meier estimator and log-rank significance test showed a significant difference between two predicted groups (i.e., short-term and long-term survival) of GBM patients (*p* = 2.9 × 10^−5^, HR = 2.76, and CI = 1.7–4.4, Figure 3B). Thus, combining radiomics (45 features while taking into account age and therapy type) with specific genomic, protein and transcriptomics features produced a significant difference between short- and long-term survival groups with *p* < 0.01. Next, we sought to identify elements which contribute most to the prediction of survival (Figure 3C). We found that the most common discriminative features of IDH1 wild type GBM are based on the combined radiomics with clinical (i.e., therapy type, age, sum of squares variance, large zone/low gray emphasis, autocorrelation, etc.) along with, genetic mutations in a limited set of genes (i.e., FUBP1, CIC, RYR2, and PIK3R1) and protein expression levels (p53 and vimentin), as shown in Figure 3C.

**Figure 4:**
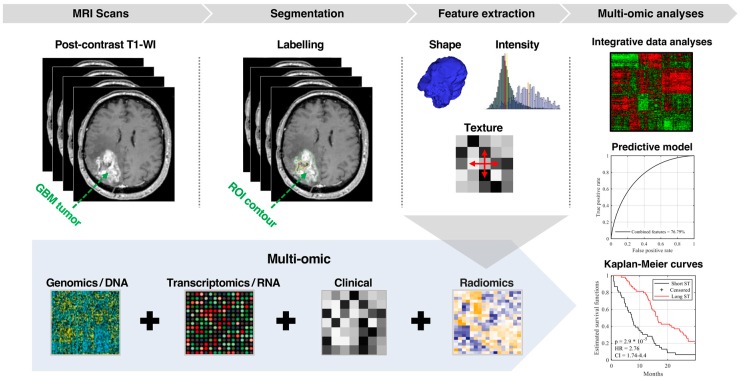
Schema of the proposed pipeline for predicting the survival outcome of GBM IDH1 wild type. (**1**) Post-contrast T1-WI MR images of 200 patients with GBM (i.e., 71 + 129 patients from TCGA/TCIA and MUHC), (**2**) identification of regions of interest (ROI) and labelling, (**3**) high-throughput radiomics features related to geometric/shape, intensity volume histogram that represents the first-order features, texture features that represent the second-order features, clinical and biological features, (4) a comprehensive dataset is synergized to develop predictive models.

To fully exploit available data (n = 200 patients), the previous analyses of AUCs in Figure 3 and Figure 4 applied an imputation strategy to include patients whose survival time is censored. To measure the impact of this factor, we repeated the multi-omics analyses using only uncensored patients (n = 189). Results can be found in Figure S4, which shows the AUC-ROC, Kaplan–Meier estimator, log-rank test p-value for the predicted survival patient groups (n = 89). In general, results observed for uncensored patients are similar to those obtained using all patients with the imputation strategy. For instance, an AUC value of 76.79% was achieved for predicting the survival groups (n = 89) using the trained multi-omics model (n = 100). Kaplan–Meier estimator and log-rank significance test showed a significant difference between two predicted groups (*p* = 0.002 HR = 1.9 and CI = 1.27–3.03). We noticed that the p-value decreased a little due to the lower number of test survival group (n = 89).

## 3. Discussion

Radiomic analysis has demonstrated its ability to draw correlations between quantitative imaging features and measures of clinical or biological characteristics [26,27,28,29,30,31,32,33,34,35,36,37]. In this context, many efforts in glioma tumor segmentation were performed to improve the radiomic and radiogenomics analysis using MRI images [31,32,38,39]. For example, radiomic features derived from peritumoral brain zone of GBM patients were found to predict short and long term of OS [34]. Likewise, the volume of necrosis has been associated with OS [15,35]. Radiomic features can also identify distinct GBM phenotypes associated with highly significant survival differences and specific molecular pathways [36]. However, few studies have comprehensively described the predictive value of radiomics upon well-annotated datasets with available MR-images, clinical measures and ‘omic’ analysis, making it hard to assess the relevance of radiomics to GBM biology and prognosis. 

In this study, we used the TCIA dataset (n = 71) and our internal MUHC dataset (n = 129) with available clinical annotations, accessible imaging and a variety of ‘omic’ analyses. We showed that (*i*) individual radiomic features analyzed in our study are not able to describe molecular subtypes (i.e., proneural, mesenchymal, neural, classical) after multiple-hypothesis correction and (*ii*) radiomic features have limited capacity to describe genetic features such as oncogene/tumor suppressor mutation status or MGMT promoter methylation. We corroborate findings that radiomics has the capacity to predict survival of patients with IDH1 wild-type GBM. However, we extend these findings and demonstrate that integration of genetic mutations, transcriptional and protein expression of a limited set of genes alongside radiomic and clinical features within a single predictive model improves performance to predict OS compared to models built from any ‘omic’ dataset alone. 

Genomic testing is becoming a routine practice in oncology, as targeted therapies are increasingly in use against disease driving oncogenes. As such, analytical techniques which determine disease characteristics are becoming fundamental for current clinical practice. We posit that radiomics offers an opportunity to increase treatment personalization at minimal additional cost. Even though integrating radiomics and along with ‘omic’ analyses is still in its early stages in oncology, our results suggest that radiomic investigations represent a complementary approach to current methods of tumor characterization [40,41] and we predict that these methods will play a significant role in enhancing the capacity for precision medicine. 

The major finding arising from this study was the stepwise improvement in predictive capacity with integration of radiomics alongside multi-omic analyses. Indeed, the greatest AUC could be achieved when considering radiomics, genomics, transcriptomics and IHC features. Interestingly, we found that the inclusion of limited genetic features (i.e., FUBP1, CIC, RYR2, and PIK3R1) and expression level of two proteins (p53 and vimentin) led to a substantial improvement in predictive models when combined along with radiomics and clinical features (i.e., age and therapy type). Interestingly, while these genes are diverse and involved in multiple pathways, there is only limited evidence suggesting an active role for them in driving differential survival in GBM patients. For example, PIK3R1 is part of the PI3K pathway where it has been demonstrated to be commonly mutated in several cancers and able to drive malignant transformation of astrocytes into a glioma-like state [42] however minimal evidence exists demonstrating a role in determining patient survival. Similarly, whilst the tumor suppressor p53 have long been investigated in cancer, the role of mutant p53 determining treatment response and predicting survival is still controversial [43,44,45]. Whilst further work is needed to demonstrate the causal role of each of these factors in driving survival differences in GBM patients, our work demonstrates that alongside radiomics, they represent a minimal signature to predict survival of GBM patients. Routine analysis of patient tumor specimens now includes multiple factors. We suggest that including the rationally identified panel of genes outlined in this study in routine practice, will provide a greater ability to predict patient survival when combined with radiomic analysis.

The poor concordance of radiomics with subtype or genomic/epigenetic features is in stark contrast to prior studies. For example, MR imaging features have been reported to be able to distinguish lesions with EGFR amplification, CDKN2A loss, and MGMT promoter methylation status [7,41,46]. Interestingly, both the expression and mutational status of genes such as EGFR has been demonstrated to vary throughout the tumor with multiple distinct clonal populations existing in concert, many of which may have very distinct genetic and phenotypic features. As such, we hypothesize that our inability to describe mutations or subtypes using radiomics is in fact because of the limited radiomic feature set and multimodality MR images. Importantly, radiomic features utilized in this study were able to identify molecular features prior to multiple-hypothesis correction, suggesting that targeted identification of individual genetic features is fully within the capacity of radiomic analysis. 

Radiomics analysis using features that depend on the manual segmentation by radiologists of ROIs like tumors can suffer from inter-reader variability [15,47]. Texture features, such as those employed in our study, are typically less affected by this bias than shape features, since they are computed over the entire segmented region and inter-reader variability mostly occurs along the boundary. In contrast, texture features are usually more sensitive to differences related to image acquisition like scanner type or parameters. In this work, we alleviated this problem by normalizing images from different sites. An alternative approach, which has been gaining in popularity, is to use deep learning models like convolution neural networks (CNNs) to learn an optimal representation directly from the images. However, training CNNs typically requires a large dataset of images which is not necessarily available. 

Our study has some limitations which could be addressed in future work. For the internal cohort, only post-contrast T1 weighted MRI sequences were available for analysis. Additionally, the findings from these limited datasets require prospective validation on a larger dataset prior to generalize clinical application. Employing multiparametric datasets (e.g., T2 weighted, FLAIR, DWI) could provide a richer set of imaging features for predicting survival. Similarly, investigating machine learning techniques like deep CNNs could potentially lead to better survival indicators.

## 4. Materials and Methods 

Figure 4 shows the flowchart of the proposed radio-genomic model to estimate the survival outcome for IDH1 wild-type GBM patients. MRI scans of contrast-enhanced T1-weighted are first acquired for GBM patients. Tumor regions of interest (ROI) are then labeled manually in each scan. Afterwards, a total of 45 radiomic features are extracted automatically from each ROI using standard shape and texture descriptors. Several analyses are considered to assess the usefulness of these features to predict overall survival (OS). First, we applied a log-rank test and Kaplan–Meier estimator to find features leading to significantly different survival curves when grouping patients based on the feature median value. To predict the short-term and long-term survival, we divided patients into two groups using the median of OS as the cut-off threshold. 

This threshold was chosen to have balanced (i.e., even-size) survival groups, and thus avoid class bias in the prediction model during both training and testing. Considering various combination of the radiomic features with genomics and proteomics, we used random forest (RF) model to classify GBM patients into groups corresponding to short (i.e., below the median OS) and long-term survival (i.e., above or equal to the median OS). The statistical significance of predicted GBM patient groups was assessed using the log-rank test and Kaplan–Meier estimator. All image manipulations, matrix calculations, significance tests, and classifications were performed in MATLAB R2017b (Math Works Inc., Natick, MA, USA).

### 4.1. Population and Data Acquisition

We retrospectively reviewed 71 GBM IDH1 wild-type obtained from The Cancer Imaging Archive (TCIA) [48], a publicly available medical image repository. Genetic, transcriptomic, and methylation data for these patients were downloaded from the TCGA repository (https://gdac.broadinstitute.org/). TCGA patients have been previously de-identified by TCGA/TCIA, and as such, no institutional review board or Health Insurance Portability and Accountability Act approval were required for our study. 

In addition, an internal cohort of 129 GBM patients from McGill University Health Centre (MUHC) was also utilized in this study. Ethical approval for this population and utilization of TMA was obtained from the MUHC Research Ethics Board (12-018 [#2442] GEN). Informed consent was obtained from all patients or their legal guardians prior to using their GBM tissue in this study. All methods were performed in accordance with the relevant guidelines and regulations. The TMA slides contained triplicate core tissue samples (1 mm in diameter) from each tumor. Four-micron-thick sections were cut and stained using a Benchmark XT immunostainer (Ventana). 

For the internal cohort, post-contrast T1-WI MR images, clinical data and various biological features including IDH1 status, MGMT methylation, and limited immunohistochemical analyses were made available for this study. Other MRI sequences like T2 weighted and FLAIR were not available for all patients in this cohort. Patient demographic information is reported in Table 1.

Since the database contains multisite data (TCGA/TCIA and MUHC), the scanner model, pixel spacing, slice thickness, and contrast varies within the selected cohort. To account for these differences, all volumes were resampled to a common voxel resolution of 1 mm^3^, for a total size of 256 × 256 × slices voxels (the slice number varying from one subject to another). Intensities within each volume were normalized to the [0,1] range. 

### 4.2. ROIs Labeling

ROIs were assigned by an expert oncologist (B.A. 25 years), manually reviewing axial gadolinium-based contrast enhancement T1-WI images of the 200 patients under blinded conditions (i.e., no clinical information). The 3D Slicer software 3.6 (http://www.slicer.org/) was used for labeling ROIs. Considering each GBM ROI separately, labeling was performed slice-by-slice from axial images to ensure accuracy and precision. 

### 4.3. Imaging Feature Extraction

Many feature extraction techniques could be used to extract the imaging features. However, we computed the most popular radiomic features from GBM ROIs. Specifically, we extracted a subset of 45 shape, intensity, and texture features with the greatest variance to minimize redundancy in descriptor features. Geometrical/shape features (porosity, fraction dimension, surface-area, and volume) encode various morphological characteristics of the ROIs that describe the geometrical variation and the tumor growth status within surrounding tissues [49,50]. Intensity features (mean, variance, skewness, kurtosis, energy, and entropy) encode characteristics of the intensity level distribution for the ROIs. For texture features, image intensities of ROIs were uniformly resampled to 32 grey-levels prior to computation in order to capture more meaningful textural patterns. We considered three different types of texture descriptors, GLCM [51], NGTDM [52] and GLSZM [53], which are described in Table S1 and listed in our previous studies [54,55]. 

### 4.4. Statistical Analysis

Radiomic analysis: The Wilcoxon test [56] was used to compare between two groups (or two classes) separated by the median radiomic feature value for enrichment of gene mutation status. The Kruskal–Wallis test was employed to examine differences in radiomic features between the four groups of GBM molecular subtypes (classical, mesenchymal, proneural, and neural). To account for multiple comparisons, all p-values obtained from significance testing were simultaneously corrected according to the Holm–Bonferroni method [23]. A threshold of *p* < 0.05 on corrected p-values was used to identify statistically significant features. 

Gene set enrichment analysis: Pre-normalized mRNA sequencing files associated with the TCIA patient cohort were downloaded from the TCGA data repository [24] and utilized in Gene Set Enrichment Analysis (GSEA) [57]. A Wilcoxon test was used to evaluate differential enrichment of functions between groups. Gene sets were considered significant if they reached a *p* < 0.05 following Holm–Bonferroni correction [23].

Survival analysis: Analysis of survival was performed by considering days to death (i.e., censorship = 1) or days to last follow-up (i.e., censorship = 0). Survival between patient groups was first compared using log-rank significance test [58] followed by Holm–Bonferroni correction [23]. Values were considered statistically significant only when *p* < 0.05 after correction. 

Multivariate analysis was undertaken by using all available features (e.g. radiomic features) as the input of random forest (RF) classifier model [59,60] to classify between short and long-term OS groups defined by the median patient survival time. RF is an ensemble method of machine learning that uses bootstrap aggregation to grow multiple decision trees (DTs) [61]. It has a built-in feature selection system and thus can handle numerous input features without having to manually exclude the irrelevant ones. Although various classifiers could be used for this task, we chose the RF with 500 DTs as it generally performs well when training data is limited and can be used to inspect the features that are most dominant in classification [59]. However, for censored patients, the time of the last follow-up only offers a lower bound on the true survival rank. This is mean that censored patients are alive at the time of data collection but have a high probability of dying in the period between the last follow-up and the maximum of overall survival. For this reason, and for considering these patients in our multivariate analysis, we employed a simple imputation strategy in which censored patients are assigned the average survival time of uncensored subjects with a time-to-death greater or equal to their own time of the last visit. A 10-fold cross-validation was used to measure the area under the ROC curve (AUC) [62]. In this strategy, training images are divided into 10 equal-sized subsets and, in each fold, one subset is put aside for testing and the remaining nine subsets are used to train the RF classifier. Performance is reported as the average AUC obtained across the 10 folds. To validate the predicted groups (i.e., patients group of short OS < median survival time versus patients group of long survival time > median survival time) by RF model, we measured the significance of the predicted survival groups by applying the log-rank test and plotting survival curves using Kaplan–Meier estimator. This strategy was employed on each of the data types (i.e., radiomic, genomics, transcriptomics, and protein expression) to find the association between data sets and survival outcome. Data integration was performed using all the available datasets as input to RF model in predicting the OS. 

Predictive features: To identify the most predictive features, we measured the increase in RF error resulting from the permutation of all considered feature values (i.e., radiomics, genetics, and proteomics) values across out-of-bag observations. Feature importance values were computed for every RF tree and averaged over the entire ensemble. These values were then normalized by dividing them using their corresponding standard deviation. Finally, the final importance of each feature was obtained by averaging these normalized values and applying the 10-fold cross-validation. Note that a positive value indicates that the feature is predictive, whereas negative values suggest that the feature has no predictive value. To compare AUC derived from the different models presented in this manuscript, we calculated significance using the chi-square test as previously described [63]. 

## 5. Conclusions

Our study underlines that radiomic features could be complimentary to biopsy-based sequencing methods to predict survival of patients with IDH1 wild-type GBM. Inclusion of limited genetic features (i.e., FUBP1, CIC, RYR2, and PIK3R1) and proteomic features (p53 and vimentin) along with radiomics features (sum of squares variance, large zone/low gray emphasis, autocorrelation) is able to predict survival of patients with IDH1 wild-type GBM. 

## Figures and Tables

**Table 1:** Demographic characteristics.

	TCGA/TCIA		MUHC Site	
	(N = 73)		(N = 132)	
	Number	%	Number	%
Age				
Median (min–max)	61(18–84)		62 (22–84)	
≤65	48	65.75	84	63.63
>65	25	34.25	48	36.36
Sex				
Female	30	41.09	73	55.30
Male	43	58.90	59	44.69
KPS				
<70	13	17.80	14	10.60
≥70	47	64.38	95	71.96
Unknown	13	17.80	23	17.42
MGMT methylation				
Methylated	24	32.87	44	33.33
Unmethylated	23	31.50	58	43.93
Unknown	26	35.61	30	22.72
IDH1				
Mutation R132H	2	2.73	3	2.27
Wild type	71	97.26	129	97.72
Extent of Surgery				
Subtotal resection	NA	-	87	65.90
Gross total	NA	-	45	34.09
Radiation treatment				
Yes	61	83.56	116	87.87
No	9	12.32	15	11.36
NA	3	4.10	1	0.75
Chemotherapy				
Yes	53	72.60	90	68.18
No	20	27.39	38	28.78
NA	0	0	4	3.03
Survival (censored)				
Median (months)	12.06		13.75	
<1year	37 (3)	50.68	59 (0)	43.70
1–4 years	31 (1)	42.46	67 (4)	50.75
>4 years	5 (2)	6.84	6 (3)	4.54

## Supplementary Materials

The following are available online at https://www.mdpi.com/2072-6694/11/8/1148/s1. Figure S1: Heatmap of p-values (negative log10 scale) comparing the distribution of radiomic features in IDH1 wild type GBM patients. First column in the heatmap represents the molecular subtypes (p-values based on the Kruskal-Wallis test to compare between four types of molecular subtype), all other columns represent the top 75 genes (p-values based on the Wilcoxon-test to compare between gene status). Figure S2: Univariate prediction of survival using radiomic features derived from FLAIR images. (A) Log-rank significance (negative log10 scale) of survival difference between individual radiomic features. (B) Multivariate prediction models integrating radiomics features derived from FLAIR with genomic features using TCIA/TCGA datasets (n = 71). Shown are area under the ROC of radiomics alone (GBMw R) or when using a combination of genetic and radiomic features (GBMw RG). (C) Kaplan–Meier estimate for the TCGA patient cohort when analyzed using the radiomics/genomics. Figure S3: Predictive capacity of all mutational features in IDH1 wild-type GBM. (A) Log-rank significance of the top 20 genes most able to predict survival in IDH1 wild-type GBM. (B) Kaplan–Meier curve for the three genes (FGD5, STAG2, FRAS1) with predictive value in IDH1 wild-type GBM. Figure S4: Validation of omics model for predicting the OS groups (i.e., short and long survival outcome) with IDH1 wild type GBM uncensored patients using 100 patient datasets for training and 89 for test (A) Area under the ROC of combined features derived from radiomics, genomics, IHC features and transcriptomics for predicting OS groups. (B) Kaplan–Meier curve of the patients’ groups that predicted their OS by omics model. Table S1: Description of features computed within the 3D volume/ROI. Table S2: Kaplan–Meier analysis of significant features for two patient groups of GBM IDH1 wild type. Table S3: Summary of gene significance. Table S4: Mutant genes. Table S5: Chi-square test to compare predicted survival groups corresponding to AUCs
