# Supplementary material for: Integration of Radiomic and Multi-omic Analyses Predicts Survival of Newly Diagnosed IDH1 Wild-Type Glioblastoma
Source: Cancers (Basel). 2019 Aug 10;11(8):1148. doi: 10.3390/cancers11081148 (PMC6721570; doi:10.3390/cancers11081148)
Supplement: Supplementary file 1 [file cancers-11-01148-s001.zip › Table S1.docx]

Table S1. Description of features computed within the 3D volume/ROI.

| **Features** | **Description** |
| --- | --- |
| **Histogram (first order features): These features are derived from the first order statistics and provide information related to the gray-level distribution of the 3D volume/ROI** | |
| Average (Mean) | The average gray level intensity |
| Variance | Measures the spread distribution about the average |
| Skewness | Measures the asymmetry of the distribution of values about the average value |
| Kurtosis | Measures the peakedness of the distribution of values in the image |
| Energy | Measures the magnitude of voxel values |
| Entropy | Describes the randomness in the image values |
| **Grey-level co-occurrence matrix (GLCM** [1]**:): These texture features use second order statistics to characterize the spatial relationship between intensity values within a 3D volume/ROI.** | |
| Angular second moment | Measures the textural uniformity that is pixel pair repetitions. It detects disorders in textures |
| Contrast | Measures the local intensity variation |
| Correlation | Describes the linear dependency of gray level values to their respective voxels in the GLCM |
| Sum of squares variance | Measures the distribution of neighboring intensity level pairs about the average of intensity level in the GLCM |
| Homogeneity | Measures the closeness of the distribution of elements in the GLCM to the GLCM diagonal |
| Sum-average | Measures the relationship between occurrences of pairs with lower intensity values and occurrences of pairs with higher intensity values |
| Sum-variance | Describes the weights elements that differ from the average value of the GLCM. |
| Sum-entropy | Represents the sum of neighborhood intensity value differences. |
| Entropy | Describes the randomness in the GLCM |
| Difference variance | Measure of heterogeneity that places higher weights on differing intensity level pairs that deviate more from the mean. |
| Difference entropy | Measure of the randomness/variability in neighborhood intensity value differences. |
| Information measure of correlation 1 | Measures the differences of randomness (entropy) |
| Information measure of correlation 2 | Measure the differences of randomness using exponential formula |
| Autocorrelation | measure of the magnitude of the fineness and coarseness of texture |
| Dissimilarity | Describes the contrast of local region |
| Cluster shade | Measure of the skewness and uniformity of the GLCM |
| Cluster prominence | Measure of the skewness and asymmetry of the GLCM |
| Maximum probability | Represents the occurrences of the most predominant pair of neighboring intensity values. |
| Inverse difference | Measures of the local homogeneity of an image |
| **Neighborhood grey-tone difference matrix (NGTDM** [2]**): These features use higher-order statistics to measure intensity differences between neighbor voxels** | |
| Coarseness | Describes the texture uniformity |
| Contrast | Describes the spatial intensity change and the overall gray level dynamic range. |
| Busyness | Measures the change from a pixel/voxel to its neighbour. |
| Complexity | An image is considered complex when it has several rapid changes in gray level intensity. |
| Texture Strength | An image is considered strength when it has a slow change in intensity but more large coarse differences in gray level intensities. |
| **Grey-level zone size matrix (GLZM** [3]**): These texture features characterize the size of uniform voxel regions, called zones.** | |
| Small zone size emphasis | Describes the fine textures. |
| Large zone size emphasis | Describes the coarse textures. |
| Low gray-level zone emphasis | Measures the distribution of lower gray-level size zones, with a higher value indicating a greater proportion of lower gray-level values and size zones in the image |
| High gray-level zone emphasis | Measures the distribution of the higher gray-level values, with a higher value indicating a greater proportion of higher gray-level values and size zones in the image. |
| Small zone / low gray emphasis | Describes the joint distribution of smaller size zones with lower gray-level values. |
| Small zone / high gray emphasis | Describes the joint distribution of smaller size zones with higher gray-level values. |
| Large zone / low gray emphasis | Describes the joint distribution of larger size zones with lower gray-level values. |
| Large zone / high gray emphasis | Describes the joint distribution of larger size zones with higher gray-level values. |
| Gray level non-uniformity | Measures the variability of gray-level intensity values in the image, with a lower value indicating more homogeneity in intensity values. |
| Zone Size Non-Uniformity | Measures the variability of size zone volumes in the image, with a lower value indicating more homogeneity in size zone volumes. |
| Zone Size Percentage | Measures the coarseness of the texture by taking the ratio of number of zones and number of voxels in the ROI |
| **Shape features** [4,5]**: describe the morphological properties of GTVs** | |
| Porosity | It is the [fraction of the volume](https://en.wikipedia.org/wiki/Volume_fraction) of voids voxels over the total volume |
| Fractal dimension | $\lim_{\varepsilon\to0} \frac{ln(N(\varepsilon))}{ln(1/\varepsilon)}$ , where *N*($\varepsilon$) is the number of cube of side $\varepsilon$ necessary to cover the ROI. |
| Volume | Represents the number of voxels in ROI. |
| Surface-area | Describes the surface of the ROI, calculated using a marching cubes algorithm^1^. |

**References**

1. Haralick, R.M. Statistical and structural approaches to texture. *Proceedings of the IEEE* **1979**, *67*, 786–804.

2. Amadasun, M.; King, R. Textural features corresponding to textural properties. *IEEE Transactions on Systems, Man and Cybernetics* **1989**, *19*, 1264–1274.

3. Thibault, G.; Fertil, B.; Navarro, C.L.; Pereira, S.; Cau, P.; Lévy, N.; SEQUEIRA, J.; MARI, J.-L. Texture indexes and gray level size zone matrix. Application to cell nuclei classification. In Proceedings of the 10th International Conference on Pattern Recognition and Information Processing, PRIP 2009; Minsk, Belarus, 2009; pp. 140–145.

4. Yang, X.; Beyenal, H.; Harkin, G.; Lewandowski, Z. Quantifying biofilm structure using image analysis. *Journal of Microbiological Methods* **2000**, *39*, 109–119.

5. Legland, D.; Kiêu, K.; Devaux, M.-F. Computation of Minkowski measures on 2D and 3D binary images. *Image Anal. Stereol* **2007**, *26*, 83–92.
