# Supplementary material for: Integration of Radiomic and Multi-omic Analyses Predicts Survival of Newly Diagnosed IDH1 Wild-Type Glioblastoma
Source: Cancers (Basel). 2019 Aug 10;11(8):1148. doi: 10.3390/cancers11081148 (PMC6721570; doi:10.3390/cancers11081148)
Supplement: Supplementary file 1 [file cancers-11-01148-s001.zip › Table S2.docx]

| **Table S2. Kaplan-Meier analysis of significant features for two patient groups of GBM IDH1 wild type** | | | | | | |
| --- | --- | --- | --- | --- | --- | --- |
| **Radiomic Features** | **Cut-off (median)** | **Median survival (months)** | | **HR** | **CI** | **p values** |
|  |  | **Above cut-off** | **Below cut-off** |  |  |  |
| **Sum square variance** | 50.1 | 15.1 | 9.7 | 0.57 | 0.42-0.77 | < 0.001* |
| **Autocorrelation** | 40.33 | 14.88 | 9.75 | 0.56 | 0.41-0.76 | < 0.001* |
| **Small zone/high gray emphasis** | 136.19 | 14.75 | 10.15 | 0.6 | 0.44-0.8 | < 0.001* |
| *p values are corrected following Holm-Bonferroni | | | | | | |
