# Supplementary material for: Integration of Radiomic and Multi-omic Analyses Predicts Survival of Newly Diagnosed IDH1 Wild-Type Glioblastoma
Source: Cancers (Basel). 2019 Aug 10;11(8):1148. doi: 10.3390/cancers11081148 (PMC6721570; doi:10.3390/cancers11081148)
Supplement: Supplementary file 1 [file cancers-11-01148-s001.zip › Table S5.docx]

| Table S5. Chi-square test to compare predicted survival groups corresponding to AUCs | | |
| --- | --- | --- |
| Site | **Features** | **p value** |
| TCIA/TCGA  n=71 | R vs. G | 1.4 × 10^-6^ |
|  | R vs. R+G | 0.01 |
|  | G vs. + R+G | 0.03 |
|  | R vs. RNA | 4.7× 10^-6^ |
|  | R vs. R+RNA | 0.08 |
|  | RNA vs. R+RNA | 9.4 × 10^-5^ |
| MUHC  n=129 | R vs. P | 2.2 × 10^-20^ |
|  | R vs. R+P | 0.3 |
|  | P vs R+ P | 0.03 |
| R: radiomics; G: genomics; P: protein expression; RNA: transcriptomics; + : combination | | |
