# Supplementary figures and images for: Integration of Radiomic and Multi-omic Analyses Predicts Survival of Newly Diagnosed IDH1 Wild-Type Glioblastoma
Source: Cancers (Basel). 2019 Aug 10;11(8):1148. doi: 10.3390/cancers11081148 (PMC6721570; doi:10.3390/cancers11081148)

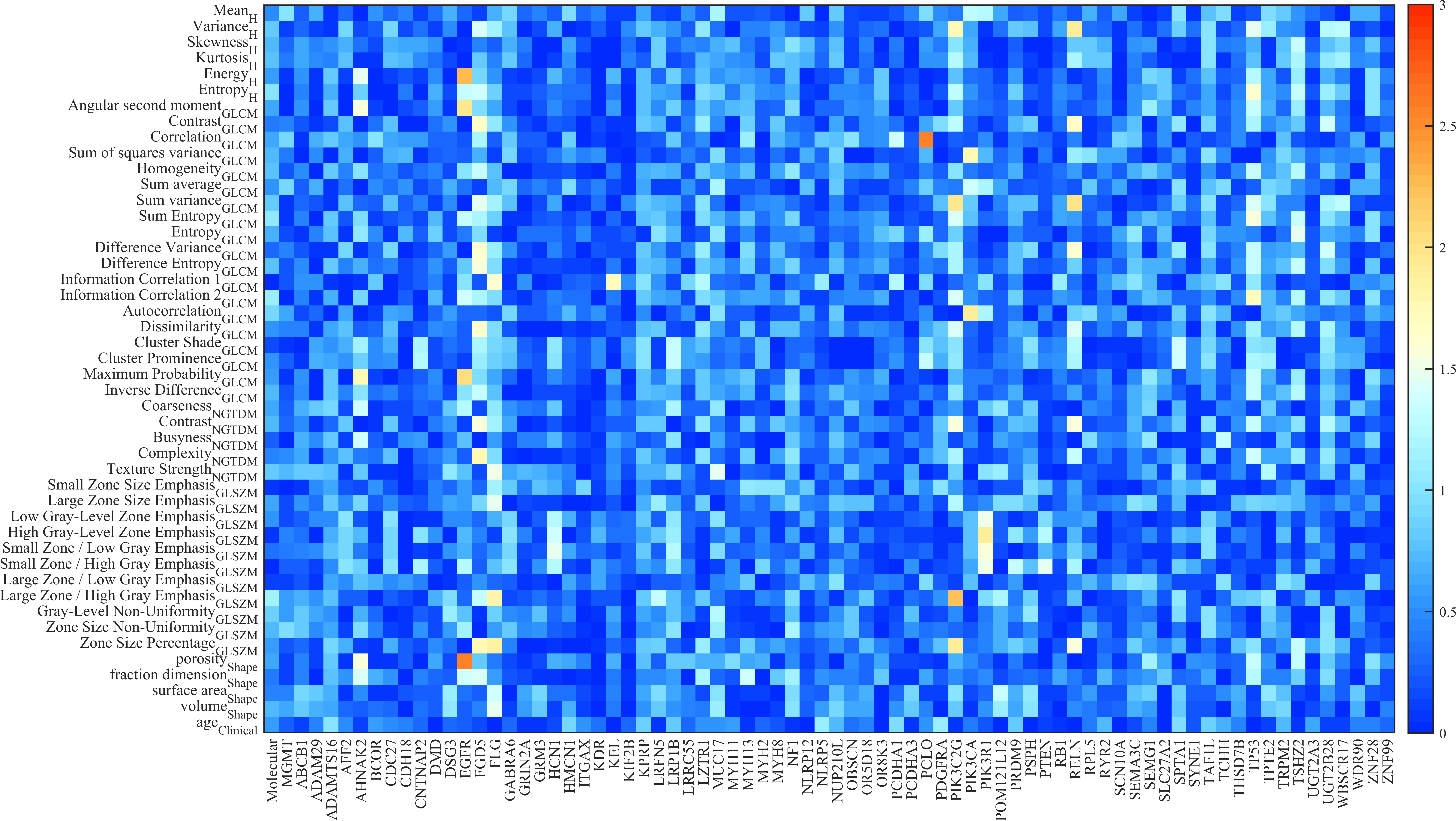

Supplement: Supplementary file 1 [file cancers-11-01148-s001.zip › Figure S1.png]

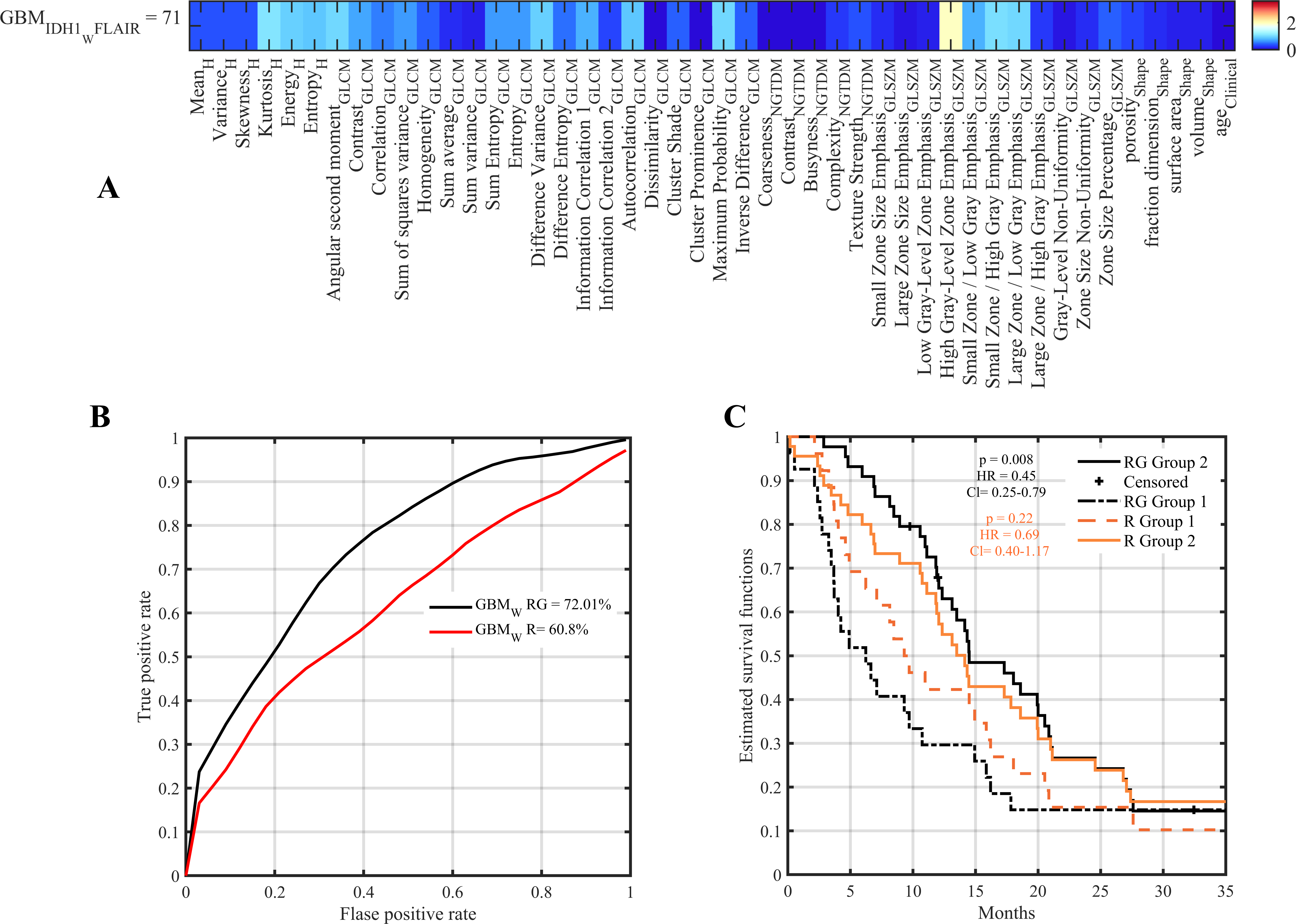

Supplement: Supplementary file 1 [file cancers-11-01148-s001.zip › Figure S2.png]

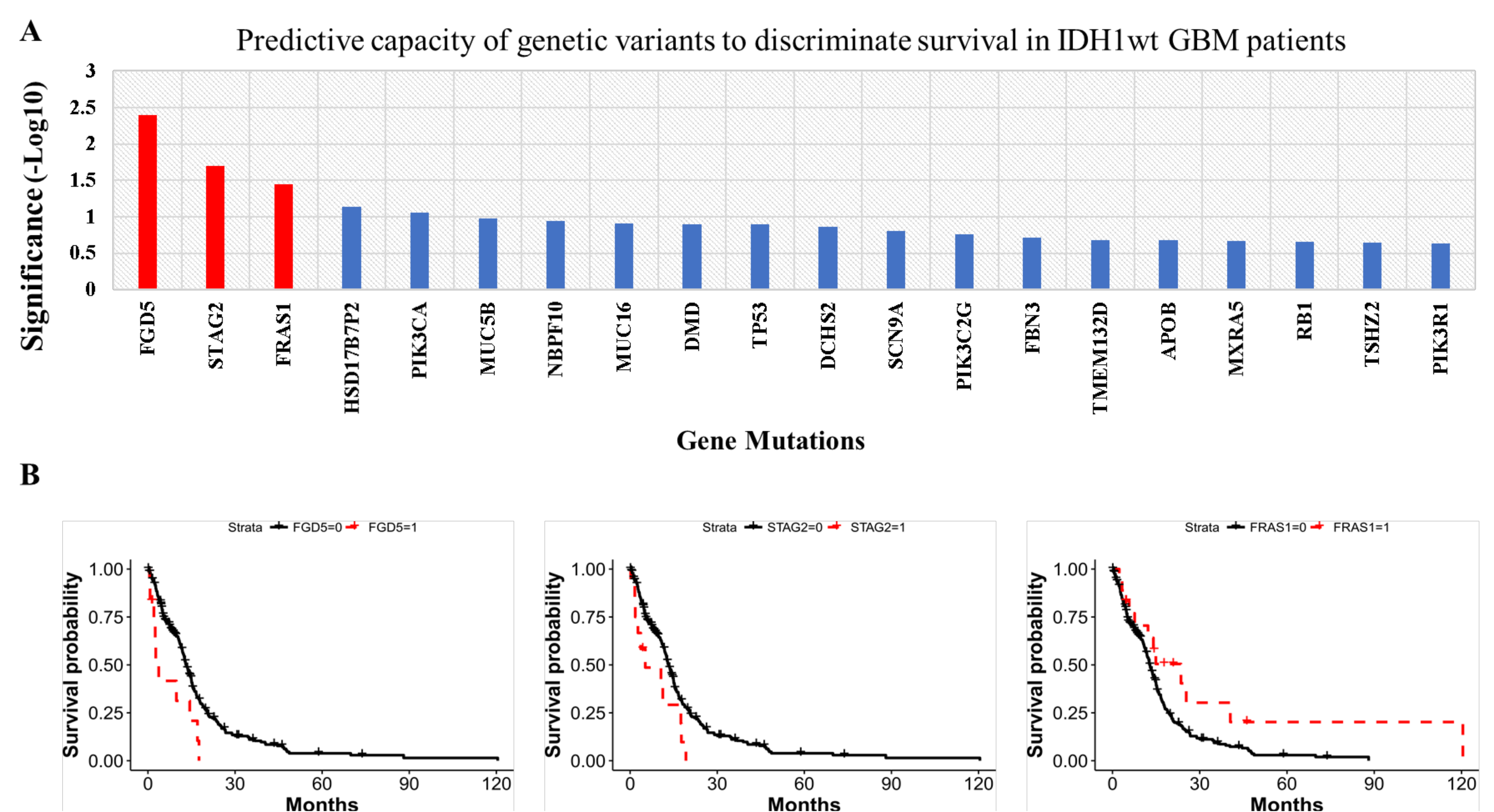

Supplement: Supplementary file 1 [file cancers-11-01148-s001.zip › Figure S3.png]

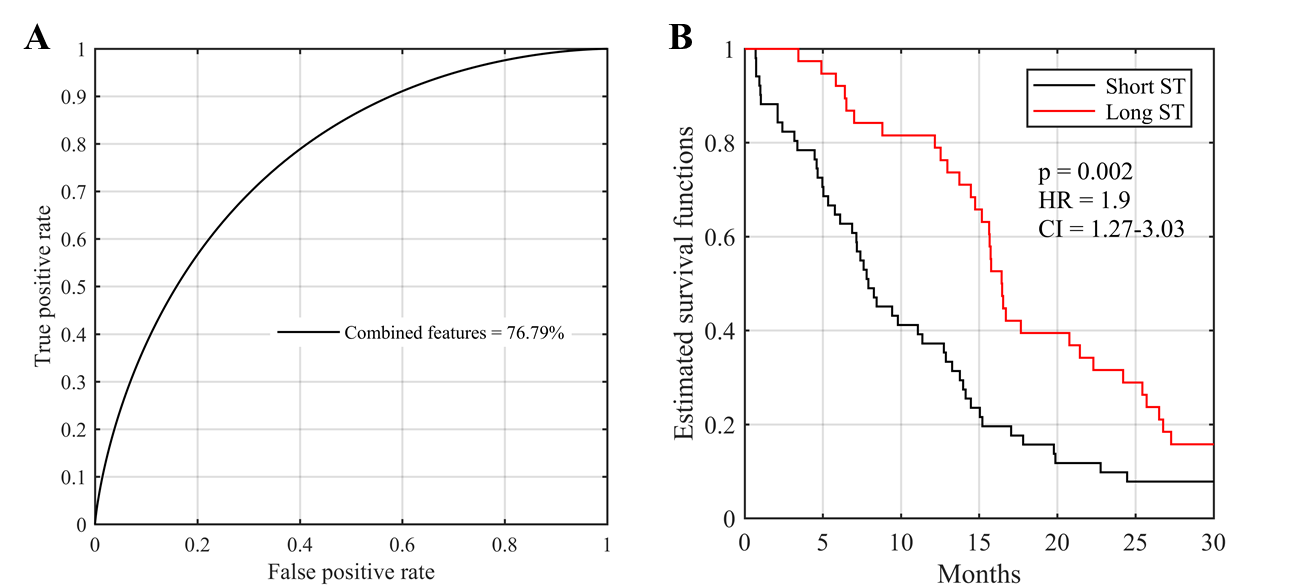

Supplement: Supplementary file 1 [file cancers-11-01148-s001.zip › Figure S4.png]
